# Co-trimoxazole–Sensitive, Methicillin-Resistant *Staphylococcus aureus*, Israel, 1988–1997

**DOI:** 10.3201/eid0909.020666

**Published:** 2003-09

**Authors:** Jihad Bishara, Silvio Pitlik, Zmira Samra, Itzhak Levy, Mical Paul, Leonard Leibovici

**Affiliations:** * Rabin Medical Center, Beilinson Campus, Petach-Tikvah, Israel; † Schneider Children’s Medical Center of Israel, Petach-Tikvah, Israel

**Keywords:** *Staphylococcus aureus*, methicillin, co-trimoxazole, resistance, susceptibility, nosocomial

## Abstract

Among bloodstream methicillin-resistant *Staphylococcus aureus* (MRSA) isolates from adult patients in a single hospital, susceptibility to co-trimoxazole increased progressively from 31% in 1988 to 92% in 1997 (p<0.0001). If also observed in other institutions, these findings should encourage the performance of a clinical trial of sufficient size to compare co-trimoxazole to vancomycin in treating MRSA infections.

Methicillin-resistant *Staphylococcus aureus* (MRSA) is a growing medical concern. During the last 2 decades, the rates of infections caused by MRSA increased among hospitalized patients in most developed countries (*1*). The aim of this study was to examine trends in antibiotic resistance of hospital-acquired bloodstream MRSA isolates from 1988 to 1997 in our institution.

## The Study

Included in the analysis were all patients >18 years of age who had hospital-acquired bacteremia caused by *S. aureus.* The study took place at Rabin Medical Center, Beilinson Campus, Petach-Tikva, Israel, a 900-bed university hospital. Our center serves an urban population of approximately 1 million persons as both a first-line and tertiary facility. A prospective surveillance of all bacteremic episodes occurring at our medical center is performed continuously and, since 1988, has been incorporated into a computerized database for bacteremia. Episodes of bacteremia are detected by daily surveillance of the microbiology laboratory records, with an annual range of 700 to 900 episodes.

Antibiotic susceptibility was tested by using the disk diffusion technique on Mueller-Hinton agar, according to the procedures established by the National Committee for Clinical Laboratory Standards (NCCLS) (*2*). Plates were incubated at 30ºC for 18 h and 40 h for methicillin (5 μg/disk) and at 37°C for 18 h for other antibiotics. Bacteremia was considered to be hospital-acquired if it appeared 48 h after admission.

During the study period, a total of 944 episodes of *S. aureus* bacteremia were documented. We found 598 (63%) hospital-acquired episodes, with an annual number of episodes ranging from 35 to 121. Among the hospital-acquired episodes, 270 (45%) were due to MRSA strains. During the recent decade, rates of resistance to methicillin were high but stable among the hospital-acquired isolates, ranging from 25% to 57%. Rates of susceptibility to co-trimoxazole among patients with hospital-acquired MRSA increased significantly from 31% in 1988 to 92% in 1997 (p=0.0001) (Figure).

**Figure:**
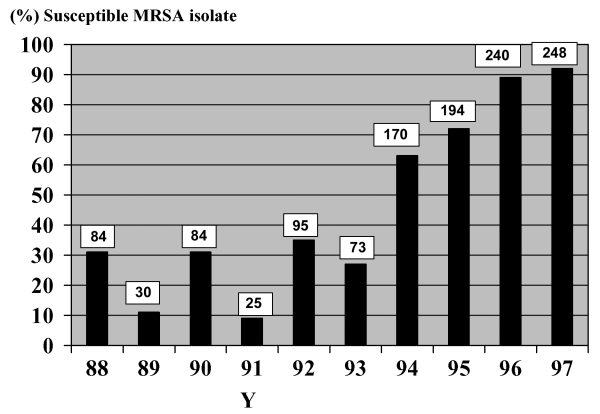
Co-trimoxazole susceptibility among methicillin-resistant *Staphylococcus aureus*. Columns indicate percentage of hospital-acquired methicillin-resistant *Staphylococcus aureus* (MRSA) susceptible to co-trimoxazole. Numbers on top of the columns are absolute numbers of hospital-acquired MRSA susceptible to co-trimoxazole.

The hospital-acquired MRSA isolates were persistently highly resistant to chloramphenicol (69% in 1988 and 100% in 1997; p=NS), gentamicin (89% in 1988 to 94% in 1997; p=NS), and ciprofloxacin (87% in 1988 to 96% in 1997; p=NS). The resistance to clindamycin (62% in 1988 to 92% in 1997; p=0.04), fusidic acid (6% in 1988 to 14% in 1997; p=0.03), and rifampicin (21% in 1988 to 76% in 1997; p=0.02) increased significantly. All isolates were sensitive to vancomycin.

## Conclusions

Our study shows that 92% of nosocomial MRSA strains were sensitive to co-trimoxazole in 1997 as compared with 31% in 1988. Several factors may have influenced the emergence of co-trimoxazole–sensitive MRSA, including the reduced usage of this drug in our institution. According to the pharmacy records, usage of co-trimoxazole in our institution decreased progressively from 28 daily doses per 1,000 hospital days in 1990 to 17 daily doses per 1,000 hospital days in 1997 (*3*). A recent multicenter report from several Belgian hospitals showed an increase in co-trimoxazole susceptibility among MRSA isolates (*4*). These findings are in contrast with trends of increasing resistance of *S. aureus* to a variety of anti-staphylococcal drugs other than co-trimoxazole, since the beginning of the antibiotic era. These trends had culminated recently with the appearance of glycopeptide resistance in hospitals and methicillin resistance in the community (*5*). Whether our findings reflect an increase of co-trimoxazole–sensitive MRSA clone/s in our institution needs further exploration. In settings where co-trimoxazole is extensively used, a substantial increase of MRSA resistance to co-trimoxazole has been observed. For example, Martin et al. described a serial cross-sectional study of resistance to co-trimoxazole among all clinical isolates of *S. aureus* and other *Enterobacteriaceae* during a 16-year period at San Francisco General Hospital (*6*). In this study, resistance to co-trimoxazole increased from 0% to 48% in *S. aureus* isolates obtained from HIV-infected patients. The authors explained this increase of resistance to co-trimoxazole by the extensive use of this drug as prophylaxis against *Pneumocystis carinii* pneumonia.

Eventually, our data may favor the use of co-trimoxazole as a potentially cost-effective antimicrobial drug for treating MRSA infections. Co-trimoxazole has been shown to be effective against MRSA both in vitro and in vivo in mice (*7*), as well as in clinical reports on meningitis, septicemia, and endocarditis (*8*,*9*). A controlled comparative trial of intravenous co-trimoxazole versus intravenous vancomycin in 101 cases of severe *S. aureus* infections in intravenous drug users was conducted by Markowitz et al. (*10*) in 1992. The authors reported 100% cure rates for either drug in MRSA infections, including bacteremia. More recently, Stein et al. showed varying degrees of success in treating with co-trimoxazole orthopedic implant infections caused by *S. aureus* (*11*). Unfortunately, this study did not distinguish MRSA from methicillin-sensitive *S. aureus* strains.

Recent in vitro data have shown good activity of co-trimoxazole against clinical isolates of vancomycin-intermediate *S. aureus* (*12*,*13*) and vancomycin-resistant *S. aureus* (*14*). In some of these cases, co-trimoxazole in combination with surgical debridement and other anti-staphylococcal drugs has been used successfully (*12*,*14*). In clinical practice, cyclical usage of co-trimoxazole and vancomycin and possible other newer anti-MRSA drugs such as oxazolidinones and streptogramins may prove of value in slowing down rates of development of antibiotic resistance in MRSA. The in vitro–presented results, if confirmed in other institutions, in conjunction with anecdotal clinical data, should encourage the performance of a clinical trial of sufficient size to compare co-trimoxazole to vancomycin in treating MRSA infections.
